# Identification of rat lung-specific microRNAs by micoRNA microarray: valuable discoveries for the facilitation of lung research

**DOI:** 10.1186/1471-2164-8-29

**Published:** 2007-01-24

**Authors:** Yang Wang, Tingting Weng, Deming Gou, Zhongming Chen, Narendranath Reddy Chintagari, Lin Liu

**Affiliations:** 1 Department of Physiological Sciences, Oklahoma State University, Stillwater, Oklahoma 74078, USA; 2 Department of Physiological Sciences, Oklahoma State University, 264 McElroy Hall, Stillwater, OK 74078, USA

## Abstract

**Background:**

An important mechanism for gene regulation utilizes small non-coding RNAs called microRNAs (miRNAs). These small RNAs play important roles in tissue development, cell differentiation and proliferation, lipid and fat metabolism, stem cells, exocytosis, diseases and cancers. To date, relatively little is known about functions of miRNAs in the lung except lung cancer.

**Results:**

In this study, we utilized a rat miRNA microarray containing 216 miRNA probes, printed in-house, to detect the expression of miRNAs in the rat lung compared to the rat heart, brain, liver, kidney and spleen. Statistical analysis using Significant Analysis of Microarray (SAM) and Tukey Honestly Significant Difference (HSD) revealed 2 miRNAs (*miR-195 and miR-200c*) expressed specifically in the lung and 9 miRNAs co-expressed in the lung and another organ. 12 selected miRNAs were verified by Northern blot analysis.

**Conclusion:**

The identified lung-specific miRNAs from this work will facilitate functional studies of miRNAs during normal physiological and pathophysiological processes of the lung.

## Background

MicroRNAs (miRNAs) include a large group of regulatory, non-coding small RNAs that measure ~22 nucleotides (nt) in length [1,2]. In animal cells, miRNAs are first transcribed from genes by the RNA polymerase, pol II, as primary miRNAs, which are then cleaved by an RNase III enzyme, Drosha, into hairpin-like precursor miRNA (pre-miRNA) [3]. The pre-miRNAs are transported into the cytoplasm with the help of a protein called Exportin 5 [4] In the cytoplasm, the pre-miRNAs are cut into double stranded RNA duplexes by another RNase III enzyme, Dicer [5]. Typically, one of the strands becomes mature miRNA and is incorporated into a RISC complex with other components for target recognition [6]. The RISC complex then binds to its target mRNA through base pairing and carries out its functions. Customarily, in the target mRNAs of an animal miRNA, there are multiple complementary sites, which correspond to the same miRNA. This cooperative action makes inhibition more efficient [7,8]. On the other hand, one miRNA usually inhibits multiple target mRNAs [9,10]. This property enables miRNA to regulate many genes in a pathway or physiological process at the same time. The action of miRNAs includes cleavage of target mRNA, translational inhibition, and mRNA deadenylation [11-15]. Several studies have shown that the degree of complementarity between the miRNA and its target determines the mode of how a miRNA works [11,13-16]. Since the discovery of the first miRNA, *lin-4 *[9], much progress has been made in the elucidation of miRNA mechanisms [1,2,9,17-20]. However, only a handful of miRNAs have been studied for their precise functions. The miRNAs in animals function in tissue development, cell differentiation, apoptosis, fat and lipid metabolism, exocytosis, stem cell division and differentiation, diseases and cancers [17,21-33]. These known functions may represent just a small part of a much bigger picture. One third of the genes in the human genome are predicted to miRNA targets [34]. With the continuing discovery of new miRNA functions, it is possible that miRNAs will be associated with the regulation of almost every aspect of cell physiology.

Spatial and temporal expression patterns of miRNAs can provide clues for their possible functions. Profile studies have already shown that many miRNAs are specifically expressed in certain organs, cell types and developmental stages. In a recent study, miRNA expression patterns among different pancreas cell types were compared [28]. One of the detected miRNAs in the study was identified as a pancreas islet-specific miRNA, which was later demonstrated to function in insulin secretion [28].

In order to study the expression profiling of miRNAs in mice and humans, several high throughput platforms have been developed. Microarrays on either membranes or slides are widely used for this purpose [35-43]. Various probe designs and labeling methods have also been utilized. Some groups have applied these microarray methods in an effort to detect the expression profiling of miRNAs in different tissues and cell types in humans and mice. Real-time PCR has also been used to detect the expression of pre-mRNAs and mature miRNAs [44,45].

The purpose of this study is to identify miRNAs that are expressed specifically in the rat lung or co-expressed in the lung and one of the five other organs in the rat. In addition, this study aims to set up a reliable, in-house miRNA microarray platform for lung research. Although the expression profiling of miRNAs in human and mouse organs has been detected by some groups, the expression in rat organs has not been extensively studied. Furthermore, relatively little is known about the detailed functions of miRNAs in the lung except lung cancers. For this study, we designed a probe set for rat miRNAs as well as human and mouse miRNAs that possess conservative sequences in the rat genome, based on the fact that miRNAs are highly conserved in animals and in plants [46-51]. The hybridizations were performed with slides printed in our laboratory. The reliability of the microarray platform was tested before other experiments were performed. As a result, our microarray allowed us to identified lung-specific miRNAs in the rat that are likely to facilitate studies of miRNA functions in the lung.

## Results

### Reproducibility and specificity of the miRNA microarray platform

**Figure 1:**
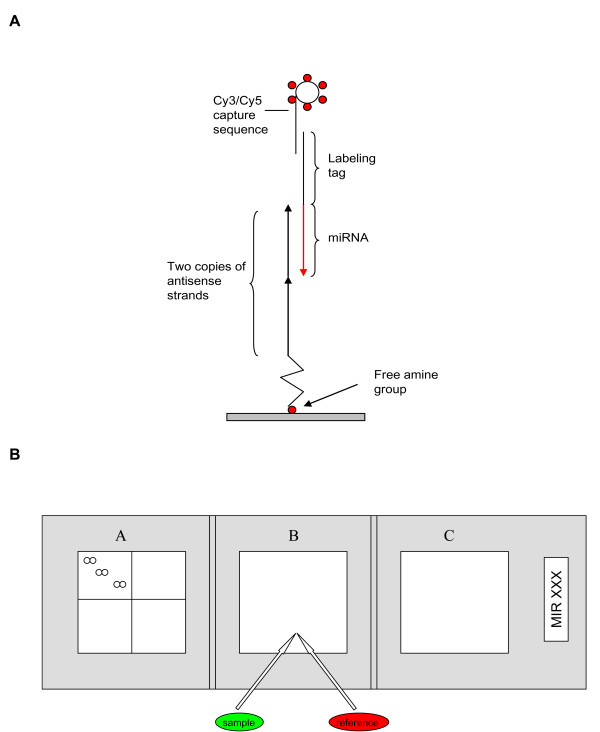
**miRNA microarray fabrication and hybridization strategy**. (A) The probes were two copies of antisense oligos of mature miRNA. The 5' ends of the probes were amino modified. The probes were linked to epoxy-coated slides covalently. miRNA samples were tagged and hybridized to the slides. (B) Each slide contained three identical blocks and each block was hybridized with two-colored paired samples. There were 6 duplicated probes for each miRNA as shown in block A. MIR xxxx, series number.

In order to evaluate the reliability of the miRNA microarray, we tested the reproducibility of the data from multiple hybridizations (Figure 1). First, equal amounts of enriched miRNAs from each of the 6 organs were pooled as the common reference. Then, miRNA from each organ (Cy3) was co-hybridized with the common reference (Cy5). The maximum and minimum probe signals in the six replicates from each hybridization were excluded from data analysis. The miRNA signal was then calculated as the geometric average of the remaining 4 probe signals. The correlation coefficient of the miRNA signals of the common references (Cy5) between two hybridizations was calculated in order to assess the reproducibility of the microarray hybridization. The results are shown in Table 1. The correlation coefficients between two hybridizations were 0.89 – 0.98. For comparison, we also used one spot per block for calculations, where the correlation coefficients were 0.83 to 0.95. The results suggest that our arrays are highly reproducible.

**Figure 2:**
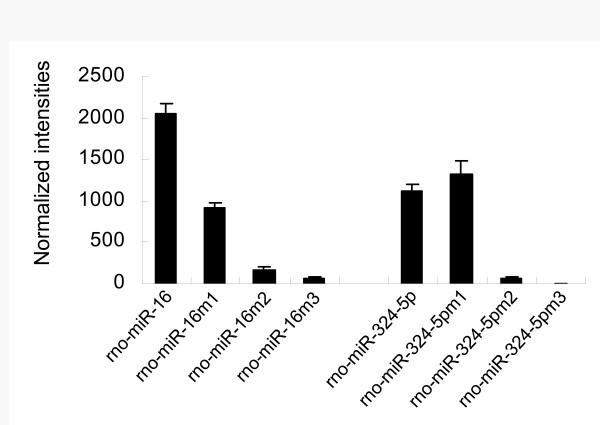
**Specificity of miRNA microarray hybridization**. The control oligos in the 3 DNA array900 microRNA direct kit were ligated with Cy5 capture sequence and hybridized onto one block. The mismatched probes had one (m1), two (m2) or three (m3) mismatched nucleotides with the corresponding control oligos. The signals from the slide were processed with the method described in Materials and Methods. Data shown were means ± S.E. from 3 hybridizations.

To test the specificity of the microarray, we used mismatched probes for two miRNAs, *rno-miR-16 *and *rno-miR-324-5p*, which were included in the control oligos provided with the labeling kit. The probes with one, two and three nucleotide mismatches were denoted with m1, m2 and m3, respectively. The microarray could not reliably distinguish those with only one mismatch. However, the signals from probes with two mismatches were significantly decreased, >20 fold less than those from probes with perfect base paring (Figure 2). From these results, we concluded that our microarray could differentiate between miRNAs with two or more nucleotide differences.

### Identification of lung-specific miRNA

**Figure 3:**
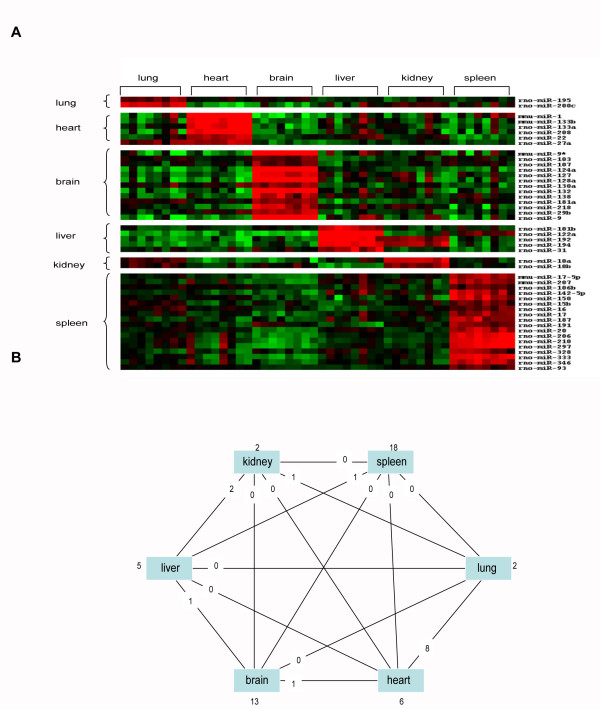
**Organ-specific microRNAs**. (A) Hot maps. The microRNA samples from 6 organs were co-hybridized with the common reference. The signals were processed as described in Materials and Methods. The normalized data were subjected to the SAM test [67]. The miRNAs that passed SAM were further tested by the HSD test (P < 0.05) to identify organ-specific miRNAs. Each column represents one hybridization and each row represents one miRNA. The figure was drawn by Treeview. Red represents positive values, green negative values, and black zero. The number of miRNAs for each organ is: Lung 2; heart 6; brain 13; liver 5; kidney 2 and spleen 18. (B) Summary of differently expressed miRNAs among 6 organs. The number beside each organ represents the number of miRNAs that are expressed significantly higher in this organ than any other organs (P < 0.05). The number on the line between any two organs is the quantity of miRNAs expressed significantly higher in these two organs than in other organs (P < 0.05).

In order to identify miRNAs that are prominently expressed in the rat lung or co-expressed in the lung and another organ, miRNA samples from 6 organs of 4 rats were co-hybridized with the common reference. A dye flip was subsequently performed. After scanning the slides, spots qualities were evaluated using the Realspot software [52]. Any miRNAs with average QI's no larger than 1 in any of the six organs were eliminated from data analysis. Out of 216 miRNAs, only 127 passed the Realspot quality test. The qualified miRNA signals were then tested by Significant Analysis of Microarray (SAM) to eliminate miRNAs that did not have significant changes between any of the six organs [53]. After the SAM test, another 21 miRNAs were eliminated, leaving 106 miRNAs for further study. Any miRNAs that were prominent in one organ or co-expressed in two organs were identified by Tukey Honestly Significant Difference (HSD) analysis (P < 0.05) (Tables 2, 3, and Figure 3). Two miRNAs (*rno-miR-195 *and *rno-miR-200c*) were identified as being expressed specifically in the rat lung. There are 5 and 3 nucleotide differences between *miR-200c*, and *miR-200a *and *miR-200b*, respectively. Therefore, our arrays were able to detect the difference between the isoforms. The numbers of the prominently expressed miRNAs in the heart, brain, liver, kidney and spleen were 6, 13, 5, 2 and 18, respectively. The numbers of co-expressed miRNAs are shown on the lines between the two organs in Figure 3. The lung had more miRNAs co-expressed in the heart than with any other organ. This finding is likely due to the relationship between the lung and the heart in organogenesis.

After HSD analysis, we calculated the organ specificity index (OSI) for organ-miRNAs that passed the SAM test. There were 2, 5, 18, 5, 1, and 16 miRNAs prominently expressed in the lung, heart, brain, liver, kidney and spleen, respectively using OSI >0.90 as a criterion (Table 2). We also noted organ specificity if the miRNA expression in one organ was at least two fold of that in all other organs. According to this two-fold definition, similar results were obtained (Table 2), showing the number of miRNAs exclusively expressed in only one of the 6 organs to be 2 (lung), 5 (heart), 14 (brain), 5 (liver), 2 (kidney), and 15 (spleen).

### Confirmation by Northern blot

**Figure 4:**
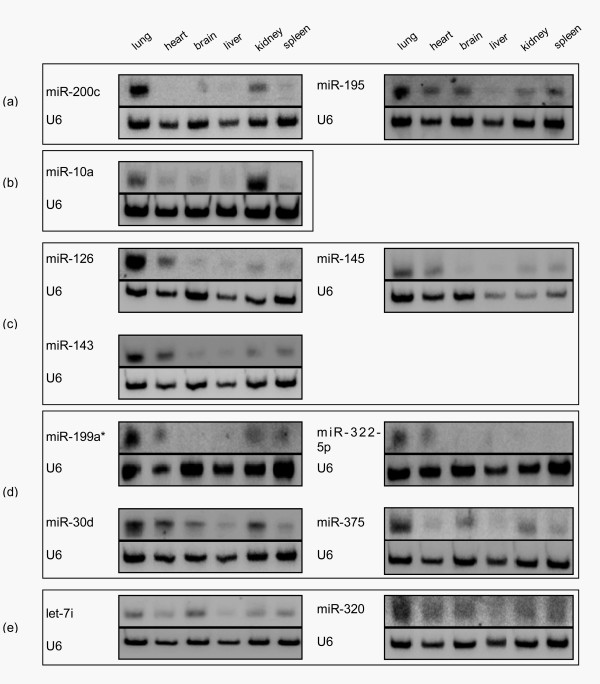
**Northern blot validation**. 15 μg total RNA was separated on a denaturing 15% PAGE gel. The ^32^P labeled probes for the microRNA were hybridized to the membrane overnight. The U6 snRNA was probed as the loading control after the hybridization of microRNA probes. (a) lung-specific miRNAs; (b) kidney-specific miRNAs; (c) co-expressed miRNAs in the lung and heart identified by HSD; (d) miRNAs highly expressed in the lung and/or the heart. (e) commonly expressed miRNAs.

**Figure 5:**
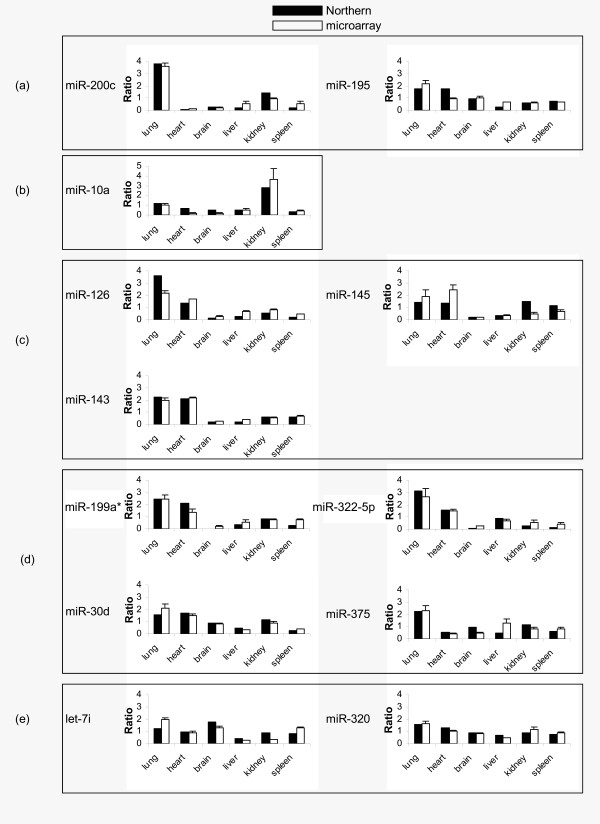
**Comparison between results from the microarray and Northern blots**. The Northern blots in Figure 4 were quantitated by the Personal Molecular Imager^® ^FX and normalized to U6. Y axes represent the normalized intensities in the Northern blots or the normalized ratios in the microarrays divided by the average of those from 6 organs. The microarray data shown are means ± S.E. from 8 hybridizations. (a) lung-specific miRNAs; (b) kidney-specific miRNAs; (c) co-expressed miRNAs in the lung and heart identified by HSD; (d) miRNAs highly expressed in the lung and/or the heart. (e) commonly expressed miRNAs.

To further investigate the reliability of our microarray data, 12 miRNAs were selected for Northern blot confirmation (Figure 4). Total RNA was extracted from the lung, heart, brain, liver, kidney and spleen from 4 rats. These were the same tissues from the rats utilized for small RNA extraction for the microarray experiment. Total RNA samples from the respective organs were pooled for the experiment. The intensities of the blots were normalized to U6 snRNA and the normalized intensities from Northern blots were compared with those from the arrays (Figure 5). Among the 12 selected miRNAs, two were lung-specific (*miR-195 *and *miR-200c*), one was kidney-specific (*miR-10a*), and three were co-expressed in the lung and heart (*miR-126*, *miR-143 *and *miR-145*) as determined by HSD, OSI and two-fold criteria. Most miRNAs had similar expression patterns from microarray analysis and Northern blots (Figure 4a–c). In a few cases, the Northern blot showed a higher expression in comparison with the microarray, including *miRNA-195 *in heart and *miRNA-145 *in kidney and spleen. We also selected additional miRNAs for verification: 3 miRNAs that had high expression in the lung and/or heart (Figure 4d and 5d) and 3 miRNAs that were expressed in most of the 6 organs (Figure 4e and 5d). Again, the results exhibited the consistency of the expression patterns between the miRNA array and Northern blot analysis. The correlation coefficients of 7 miRNAs between microarray and Northern blot were higher than 0.9. Although there were few discrepancies, it is clear that the microarray data agreed with the Northern blot data.

## Discussion and conclusion

In this study, we designed a miRNA microarray system in our laboratory and tested its reliability. This method was used to compare the miRNA expression patterns of six different rat organs, namely the lung, heart, brain, liver, kidney and spleen. We identified 2 miRNAs (*miR-195 *and *miR-200c*) that were distinctively expressed in the rat lung, 8 miRNAs that were co-expressed in the lung and heart and 1 miRNA that was co-expressed in the lung and kidney. The reliability of our microarray was confirmed by the high consistence between the microarray and Northern blot analysis.

Several groups have developed miRNA microarray platforms to detect the profiling of miRNAs. However, our platform has several unique features. First, we used two-channel co-hybridization. We also pooled all of the samples as the common reference. Additionally, we performed the dye swap to eliminate the effect of dye bias. The approaches are similar to those used in DNA microarrays. Conversely, most of the other platforms use a single channel platform. Some groups have utilized synthesized oligos as the common reference in their microarrays [37,43], but the DNA-RNA hybridization may differ from DNA-DNA hybridization. Second, we have 6 replicate spots in our microarray, making us able to exclude the maximum and minimum values while being able to calculate the geometric average signal from the remaining 4 replicates. This method significantly increased the reproducibility of the data. Third, we printed slides in-house with 3 identical blocks in each slide. This procedure allowed us to hybridize 6 samples (3 pairs) on a single slide. We have previously demonstrated that this approach significantly reduces the variance and increases the efficiency [54].

When comparing our data with previous studies, there are similarities as well as differences. For example, only one of the miRNAs we identified as lung-specific, *miR-200c*, has been reported by other groups [37]. Also, *miR-195 *was previously reported to be expressed higher in the spleen than in the lung [37], but both our microarray and Northern blot results show *rno-miR-195 *expressing much higher in the lung than in the spleen. A few factors could have lead to these discrepancies. First, the miRNAs printed on our microarray slides were different from others. Some platforms did not contain all of the 217 miRNAs that we printed on our slides. For example, *mmu-miR-375 *is a newly identified miRNA expressed highly in the lung. This particular miRNA was not included on microarray slides in the other studies. Therefore, our study is supplemental and concurrent with other miRNA profiling studies. Second, the sample origins, hybridization conditions, and data analysis methods were different among different research groups. Our miRNA samples were extracted from rat organs and those of other groups were extracted from human and mouse organs. Although this may cause some differences, most miRNAs identified thus far are conserved among species. The use of different hybridization conditions and normalization methods may have also caused some inconsistencies in sensitivity and specificity. However, the methods and conditions used in our study are highly reproducible. Third, some miRNAs that were lung-specific were not highly expressed. This may have caused a disagreement with other microarray platforms. Indeed, when we compared the highly expressed miRNAs that we found in the rat brain to those identified by other groups, 11 out of 13 were consistent with others [35,37,43,55], suggesting that our results are reliable and comparable to other miRNA microarray platforms.

There are some differences between the results from the microarray and the Northern blots. The most obvious reason for the variation was that the hybridization conditions and the normalization methods were different between the two. The hybridization temperatures and buffer affected the sensitivity and specificity of the assays. The assumption for the microarray normalization was that the total amount of miRNA was consistent between samples from different organs. Realistically, this assumption was not true in some cases. The signals from the Northern blots were normalized to the signals of U6 snRNA with the assumption that the amount of U6 snRNA was the same as the amount of total RNA from the different organs. It is virtually impossible to provide the exact same amount of RNA between samples.

Among the 12 miRNAs confirmed by the Northern blots, none has a known function except *mmu-miR-375 *[28]. The expression of *mmu-miR-375 *was reported to be limited to the pancreatic β cells, although we also detected it in the rat lung. It has been reported to regulate the secretion of insulin. Neither the secondary signals nor the actin filament network are affected by *mmu-miR-375*. *Mtpn *was validated as a target gene of *mmu-miR-375*. *Mtpn *was reported to form a complex with CapZ which regulates actin polymerization [56]. In the lung, the alveolar epithelial type II cells secrete surfactant through exocytosis, which helps to reduce the surface tension of the alveolar sacs and facilitate the normal function of gas exchange. The mechanism of the secretion of surfactant in the lung is similar to that of the secretion of insulin in the pancreas. We suspect that *mmu-miR-375 *works in both of these exocytosis processes. We may find some hints as to the mechanism of exocytosis in the lung if we find more targets of *miR-375 *or any of the components that interact with these targets.

Two well-known miRNAs, *miR-1 *and *miR-133*, have highly specific expression in cardiac and skeletal muscle tissue [55]. In our study, we also identified these miRNAs as having heart-specific expression. These two miRNAs are clustered together in the mouse genome and both of them modulate muscle proliferation and differentiation. *miR-1 *promotes myogenesis by targeting histone deacetylase 4 (*HDAC4*), while *miR-133 *promotes myoblast proliferation by inhibiting serum response factor (*SRF*) [57].

A brain-specific miRNA, *miR-9*, has been identified by our microarray as well as by microarrays from other groups. It has been shown to affect neural lineage differentiation in ES cells. STAT3, which is a member of the signal transducer and activator of transcription family, is believed to be involved in this function [27]. In presenilin-1 null mice, *miR-9 *has been shown to be down-regulated, leading to severe brain developmental defects [38].

The liver-specific miRNA, *miR-122*, likely modulates the hepatitis C virus by facilitating replication of the viral RNA. Mutational analysis and ectopic expression studies have revealed that *miR-122 *interacts with the 5' non-coding region of the viral genome [58]. This suggests that *miR-122 *may be a target for antiviral interaction [58]. In addition, *miR-122 *is a key regulator of cholesterol and fatty-acid metabolism in the adult liver by regulating plasma cholesterol levels, fatty-acid oxidation, hepatic fatty-acid synthesis as well as cholesterol synthesis [22].

Among the spleen-specific miRNAs identified, five of them belong to the *mir17 *miRNA cluster, which comprise *miR-17*, *miR-18*, *miR-19a*, *miR-19b*, *miR-20*, *miR-25*, *miR-92*, *miR-93*, *miR-106a*, and *miR-106b *[59]. Among these 5 miRNAs, *miR-17-5p*, *miR-17 *and *miR-20 *belong to one of the *mir17 *microRNA clusters, the *mir-17–92 *cistron, which is one with well characterized cancer association. The *mir-17–92 *polycistron is located at 13q31, a genomic locus that is often amplified in cancers. The substantial increase in the expression of microRNAs from this cistron has been reported in human B-cell lymphomas and human lung cancers [60,61]. However, the prominent expression and function of these miRNAs in the spleen are not known.

There are few studies concerning the functions of miRNAs in the lung. Several recent studies have given rise to a great interest in this field of research. The reduction in the expression of *let-7 *in human lung cancers is correlated to increased death rates in patients [62]. Experimentally, over-expression of *let-7 *can inhibit lung cancer cell growth *in vitro*. This discovery shows that *let-7 *may have potential clinical value in treating lung cancers. Inactivation of Dicer results in the defect of epithelial branching [63]. This defect is independent of the requirement for Dicer in cell survival and does not stop the epithelial growth [63]. In the E11.5 lung, Ago1 and Ago2 are enriched in the branching regions, which undergo the most dynamic changes during lung remodeling. This discovery suggests that miRNAs regulate processes responsible for the biogenesis of the lung [64]. Another study shows that the decrease in Dicer expression is associated with the poor prognosis in lung cancer patients [65]. The miRNA expression profiles in lung cancers correlate with the prognosis of lung adenocarcinoma patients [66].

In summary, we designed a reliable miRNA microarray platform that is low in cost and easy to update with highly reproducible results. The expression profiling of microRNAs in 6 rat organs was detected with this platform. The expression patterns of lung-specific and lung co-expressed microRNAs were confirmed by Northern blot analysis. Our platform adds to the implementation of detecting microRNA profiles, as no other microarray platform has been made for the detection of rat microRNA profiles. Furthermore, our microarray platform contains several recently discovered miRNAs, making it supplementary to other platforms. When applied, our study of the expression patterns of miRNAs in the lung should shed light on the functions of miRNAs in lung physiology as well as lung pathophysiology.

## Methods

### Microarray fabrication

217 mature miRNA sequences were downloaded from the miRNA registry (Wellcome Trust Sanger Institute). 177 of these were from rat, and 40 non-redundant conservative ones were from human or mouse. Sequences of some of these human and mouse miRNAs did not match their corresponding sequence in the rat genome exactly and were modified in accordance with those in the rat genome. The probes for the miRNAs had two copies of antisense sequences (34–50 nt) (Figure 1A). Probe sets, which contained 5' amino modified C6 oligos were synthesized by Sigma-Genosys (Woodlands, TX) at 100 μM concentration and suspended in 3 × SSC buffer. The oligos were diluted to 25 μM with 3 × SSC prior to use. The probes were then printed onto epoxy-coated slides (CEL Associates, Pearland, TX) with an OmniGrid 100 array (GeneMachine, San Carlos, CA) at 65% humidity and then incubated for 48 hours at the same humidity. Each slide contained three identical blocks in a landscape orientation. Within each block every probe was printed 6 times in 3 separate pairs (Figure 1B). The oligo set also contained one probe for U6 and 3 probes for tRNAs as positive controls as well as one probe for a plant miRNA as a negative control.

### Tissue sample and small RNA extraction

Four male Sprague-Dawley rats (200 g, Charles River Laboratories, Inc., Wilmington, MA) were anaesthetized. The six organs (lung, heart, brain, kidney, liver and spleen) were collected and powdered in liquid nitrogen. Small RNA was enriched from the powdered samples from the 6 rat organs using the *mir*Vana™ microRNA isolation kit from Ambion (Austin, TX), according to the manufacturer's protocol. First, 200 mg powder was homogenized in 2 ml Lysis/Binding Buffer. Then, one-tenth of the volume of miRNA homogenate additive was added to the homogenate and a volume of acid-phenol: chloroform was used to extract RNA. One-third volume of 100% ethanol was added to the aqueous phase, and the sample was passed through a filter cartridge. Two-thirds volume of 100% ethanol was then added to the filtrate, and the sample was passed through a second filter cartridge. The second filter cartridge was subsequently washed once with wash solution 1 and then twice with Wash Solutions 2 and 3. Afterwards, the small RNA was eluted with 95°C nuclease-free water. Total RNA for Northern blots was also extracted from these organs by the aforementioned protocol. Following organic extraction, one and one-forth volumes of 100% ethanol was added to the aqueous phase. The lysate/ethanol mixture was then passed through a filter cartridge. The cartridge was washed, and the total RNA was eluted with water as described above. The concentration of RNA was determined by a NanoDrop ND-1000 Spectrophotometer (NanoDrop Tech., Rockland, DE). The quality of enriched small RNA was determined on a denaturing 15% polyacrylamide gel, and the quality of total RNA was tested on a 1% agarose gel.

### miRNA labeling and microarray hybridization

The labeling and hybridization of miRNA were performed with the 3 DNA array900 miRNA direct kit (Genisphere, Hatfield, PA), according to the manufacturer's protocol. Enriched small RNA (120 ng) was used for each hybridization. First, the miRNA was tailed with poly A by PAP enzyme (poly (A) polymerase). Then the capture sequence was ligated to the tailed miRNA. Tagged miRNA was purified with the MinElute PCR Purification Kit (Qiagen, Valencia, CA). All of the small RNA samples were separately labeled with Cy3 or Cy5 capture sequence. After labeling and purification, equal amounts of small RNA from all the samples, labeled with the same dye, were pooled together as a common reference. The hybridization was performed as previously described [54]. To each block, one labeled sample was hybridized along with a common reference labeled with the other dye. Dye-swap was performed to eliminate dye bias. Tagged miRNA hybridizations were performed at 52°C overnight, and then the slide was washed 15 min in pre-warmed 2 × SSC, 0.2% SDS, followed by 12 min in 2 × SSC at room temperature and finally for 12 min in 0.2 × SSC at room temperate. The 3 DNA hybridization was performed at 62°C for 4 h, and then the slide was washed and dried.

### Microarray data analysis

The hybridized slides were scanned with ScanArray Express (PerkinElmer Life and Analytical Sciences, Boston, MA), and the images were analyzed with GenePix 5.0 pro (Axon Instruments, Inc. Union City, CA). The signal from each spot was normalized to the average signal of the whole block. The highest and lowest signals from the 6 identical probes in the same block were excluded from the data analysis. The geometric average of the other 4 signals was considered to be the signal of that particular miRNA. The ratio of the sample signal to the reference signal was log2 transformed. A quality test was performed with the software, Realspot, developed in our laboratory [52]. The miRNAs with an average quality index of <1 were filtered. The miRNAs that passed the quality test were analyzed with SAM (Significant Analysis of Microarray) in order to choose miRNAs that were significantly changed between different organs (q < 0.01) [53]. These miRNAs were then subject to the Tukey Honestly Significant Difference (HSD) test (p < 0.05) [54]. The organ specificity index (OSI) was also used to determine the relative specificity of miRNA in organs. The OSI was defined as the correlation coefficient of miRNA expression between a miRNA and a putative miRNA whose expression levels were given the value of 1,000 in prominent organs and a value of 0 in other organs [54].

### Northern blot analysis

Total RNA from the same organ was pooled together. The probe sequences were exactly the same as the antisense sequences to miRNAs except that those with sequences which started with C were capped with G or T at the 5' end to increase ^32^P labeling efficiency. RNA samples were denatured at 95°C for 4 minutes. 15 μg total RNA was separated on a 15% denaturing PAGE gel at 100 V for 2 h in 1 × TBE buffer. The RNA was then transferred to a Hybond-N^+ ^membrane (Amersham, Piscataway, NJ) using a Trans-blot SD semi-dry transfer cell (Bio-Rad, Hercules, CA) at 20 ~ 25 V for 1 hour using 0.25 × TBE as a transfer buffer. Membranes were UV crosslinked with a 120 mJ burst and then baked at 80°C for 1 hour. For each sample, 20 pmol antisense oligonucleotide probes were end labeled by γ^32^P dATP (>7000 Ci/mmol, MP Biomedical, Irvine, CA) with T4 polynucleotide kinase (NEB, Ipswich, MA) for 4 hours at 37°C. The reactions were stopped with 2 μl 0.5 M EDTA. The probes were then purified with a G-25 MicroSpin column (Amersham). Pre-hybridization and hybridization were carried out at 30°C using ULTRAhyb-Oligo hybridization buffer (Ambion), according to the manufacturer's manual. After hybridization the membranes were washed twice with 2 × SSC 0.5% SDS for 30 minutes at 30°C. The membranes were then exposed to a phosphor screen overnight and scanned with the Personal Molecular Imager^® ^FX (Bio-Rad). U6 was probed as a loading control and only exposed to the phosphor screen for 5 to 10 minutes.

## Authors' contributions

YW carried out microRNA microarray printing, hybridization, data analysis and northern blot and drafted the manuscript. TW participated in the array printing and data analysis. DM participated in northern blot analysis. ZC participated in data analysis. NRC participated in sample collection. LL conceived of the study, and participated in its design and coordination and helped to draft the manuscript. All authors read and approved the final manuscript.

## Figures and Tables

**Table 1:** The correlation coefficients of common reference signals between two hybridizations

	lung	heart	brain	liver	kidney
heart	0.984393				
brain	0.980202	0.973659			
liver	0.973702	0.97738	0.965574		
kidney	0.96804	0.971573	0.965264	0.978328	
spleen	0.895349	0.893531	0.92511	0.917216	0.925989

Cy3-labeled organ and Cy5-labeled common reference (pooled samples from 6 organs) were co-hybridized. The correlation coefficients were calculated from the log2 transformed average signals of the common reference between two hybridizations.

**Table 2:** Organ-specific microRNAs identified by HSD test, OSI test and two-fold criteria.

**Lung (2)**			**Heart (6)**			**Brain (13)**			**Liver (5)**			**Kidney (2)**			**Spleen (18)**		
	OSI	2 fold		OSI	2 fold		OSI	2 fold		OSI	2 fold		OSI	2 fold		OSI	2 fold

mo-miR-195	0.957		mmu-miR-1	0.999		mmu-miR-9*	0.999		mo-miR-101b	0.998		mo-miR-10a	0.975		mmu-miR-17-5p	0.983	
mo-miR-200c	0.973		mmu-miR-133b	0.998		mo-miR-103	0.902		mo-miR-122a	1.000		mo-miR-10b	0.899		mmu-miR-207	0.973	
			mo-miR-133a	0.999		mo-miR-107	0.967		mo-miR-192	0.956		mo-miR-200b†	0.900		mo-miR-106b	0.911	
			mo-miR-208	0.999		mo-miR-124a	1.000		mo-miR-194	0.914					mo-miR-142-5p	0.976	
			mo-miR-22	0.975		mo-miR-127	0.995		mo-miR-31	0.994					mo-miR-150	0.992	
			mo-miR-27a	0.886		mo-miR-128a	1.000								mo-miR-15b	0.895	
						mo-miR-128b†	0.964								mo-miR-16	0.857	
						mo-miR-130a	0.940								mo-miR-17	0.971	
						mo-miR-132	0.999								mo-miR-187	0.990	
						mo-miR-137†	0.958								mo-miR-191	0.944	
						mo-miR-138	0.980								mo-miR-20	0.982	
						mo-miR-181a	0.925								mo-miR-206	0.998	
						mo-miR-218	0.982								mo-miR-210	0.997	
						mo-iR-221†	0.916								mo-miR-297	0.997	
						mo-miR-29b	0.981								mo-miR-328	0.980	
						mo-miR-29c†	0.906								mo-miR-333	0.997	
						mo-miR-9	1.000								mo-miR-346	0.995	
															mo-miR-93	0.944	

The numbers in the parenthesis are the quantities of the microRNAs identified by HSD test. The listed miRNAs were identified by HSD except those marked by "†". The symbol, "" indicates the organ-specific miRNAs that were identified by two-fold criteria. A OSI of >0.9 was considered as organ-specific.

**Table 3:** miRNAs co-expressed in two organs

**lung-heart**		**lung-brain**		**lung-kidney**		**heart-brain**		**heart-spleen**		**brain-liver**		**liver-kidney**		**liver-spleen**	
	2-fold		2-fold		2-fold		2-fold		2-fold		2-fold		2-fold		2-fold

mmu-miR-322-5p†	√	rno-miR-125a†	√	rno-miR-10a		rno-miR-29b	√	mmu-miR-1†	√	rno-miR-128b	√	rno-miR-192	√	rno-miR-122a	√
mo-miR-126	√					mo-miR-29c†	√					rno-miR-194	√		
mo-miR-143	√														
mo-miR-145	√														
mo-miR-23a	√														
mo-miR-23b	√														
mo-miR-24															
mo-miR-27a	√														
mo-miR-27b															

The listed miRNAs co-expressed in two organs were identified by HSD except those marked with "†". The symbol "√" indicates the miRNAs that were identified by 2 fold criteria.
